# Copper- and Silver-Containing Heterometallic Iodobismuthates: Features of Thermochromic Behavior

**DOI:** 10.3390/ijms24087234

**Published:** 2023-04-13

**Authors:** Irina A. Shentseva, Andrey N. Usoltsev, Nikita A. Korobeynikov, Taisiya S. Sukhikh, Vladimir R. Shayapov, Maxim N. Sokolov, Sergey A. Adonin

**Affiliations:** Nikolaev Institute of Inorganic Chemistry, Siberian Branch of Russian Academy of Sciences, Lavrentieva St. 3, 630090 Novosibirsk, Russia

**Keywords:** halometallates, thermochromism, bismuth, coordination polymers

## Abstract

Nine heterometallic iodobismuthates with the general formula Cat_2_{[Bi_2_M_2_I_10_}] (M = Cu(I), Ag(I), Cat = organic cation) were synthesized. According to X-ray diffraction data, their crystal structures consisted of {Bi_2_I_10_} units interconnected with Cu(I) or Ag(I) atoms through I-bridging ligands, forming one-dimensional polymers. The compounds are thermally stable up to 200 °C. Optical band gaps (E_g_), estimated at room temperature via diffuse reflectance measurements, range from 1.81 to 2.03 eV. Thermally induced changes in optical behavior (thermochromism) for compounds **1**–**9** were recorded, and general correlations were established. The thermal dependence of E_g_ appears to be close to linear for all studied compounds.

## 1. Introduction

Anionic halide complexes of p-block metals are of particular interest because of their high structural diversity, ranging from discrete blocks to three-dimensional systems [1,2,3], and number of peculiar physical properties, including ferroelasticity and ferroelectricity [4,5,6,7], photocatalytic activity [8,9,10,11,12] and optoelectronic properties [13,14,15,16], etc. In particular, recent developments in perovskite halide photovoltaics, primarily based on methylammonium lead iodide (MAPI) [17], have led to power conversion efficiencies in these materials now exceeding 25%, making this technology theoretically suitable for wider implementation [18]. Furthermore, there are significant prospects for the further development of this area, as evidenced by the rapid growth in research articles in highly ranked scientific journals. However, the high toxicity of lead and the poor long-term stability of Pb-based photovoltaic materials are key problems, prompting researchers to explore other, more stable and less toxic materials as possible alternatives, utilizing halometallates of other *p*-elements, such as Sn, Bi, Sb and Te [3].

Amongst the wide variety in the studied materials, prepared through various approaches, one promising method for altering electronic properties and developing new materials should be particularly emphasized: the combination of *p*- and *d*-elements (*d*-elements typically being copper and silver) in the anion. According to this strategy, heterometallic copper- and silver-containing iodobismuthates with zero-dimensional [19,20,21,22,23,24], one-dimensional [20,25,26,27,28] (Figure S1) or two-dimensional [29,30] anionic motifs have been characterized previously. However, the overall number of known heterometallic iodometallates remains lower than that of homometallic ones, but this trend can change, as recent works clearly demonstrate [27,31,32,33,34].

Thermochromism is a reversible or irreversible change in the color of compounds when they are heated or cooled. This property is considered to be common for halometallates, as demonstrated in several previous reports on Bi(III) [22,35,36,37,38] and halide complexes of Ag and Cu [39]. However, these reports have two significant drawbacks: (1) almost all studies were performed on a single complex, rather than a series, and (2) the spectra measurements were performed at one or two temperatures, making a detailed evaluation of temperature dependencies impossible. Recently, we investigated the thermochromic behavior of a series of Te(IV) mononuclear halometallates [40] with varying substituted pyridinium-type cations, introducing a more advanced approach to such work, which involved multi-temperature spectra measurements. Continuing our work with heterometallic iodobismuthates templated by functionalized Py-based cations [41,42] and drawing on our experience with thermochromic bromotellurates [40], we decided to systematically study the potential thermochromic properties of the entire series of compounds.

**Figure 1:**
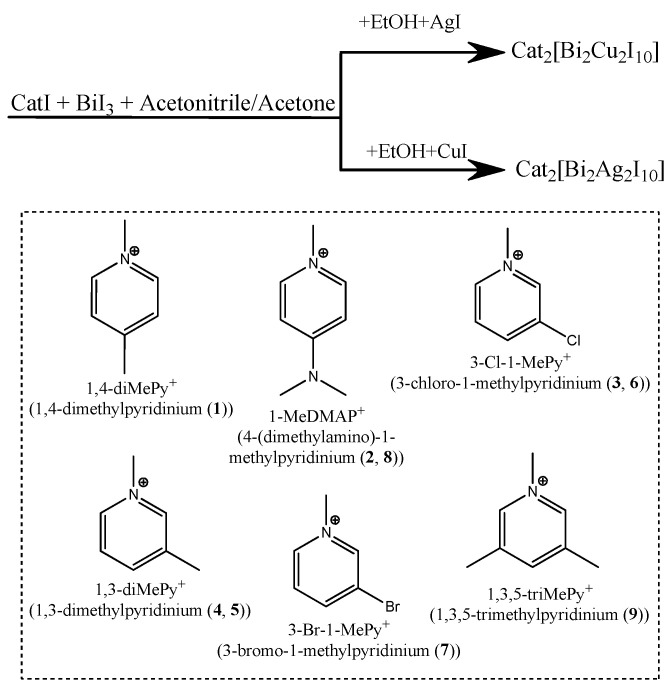
Schematic for the structures of cations for compounds **1**–**9** and their general routes of synthesis.

Herein, we report a systematic study of four copper heterometallic iodobismuthates. (Cat)_2_[Bi_2_Cu_2_I_10_] (Cat = 1,4–diMePy^+^ (**1**), 1–MeDMAP^+^ (**2**), 3–Cl–1–MePy^+^ (**3**), 1,3–diMePy^+^ (**4**)) and five silver heterometallic iodobismuthates ((Cat)_2_[Bi_2_Ag_2_I_10_] (Cat = 1,3–diMePy^+^ (**5**), 3–Cl–1–MePy^+^ (**6**), 3-Br–1–MePy^+^ (**7**), 1–MeDMAP^+^ (**8**), 1,3,5–triMePy^+^ (**9**)) (Figure 1). The compounds were analyzed using X-ray diffraction techniques, TGA and diffuse reflectance spectroscopy. Optical spectra of compounds **1**–**9** were measured in a temperature range of −170–25 °C, and temperature dependencies of the band gap were established.

## 2. Results and Discussion

**Figure 2:**
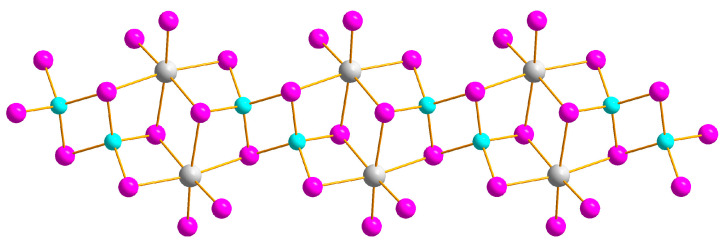
{[Cu_2_Bi_2_I_10_]_n_}^2n−^ anion in compounds **1**–**4**. Here and below: Bi grey, I purple, Cu turquoise.

**Figure 3:**
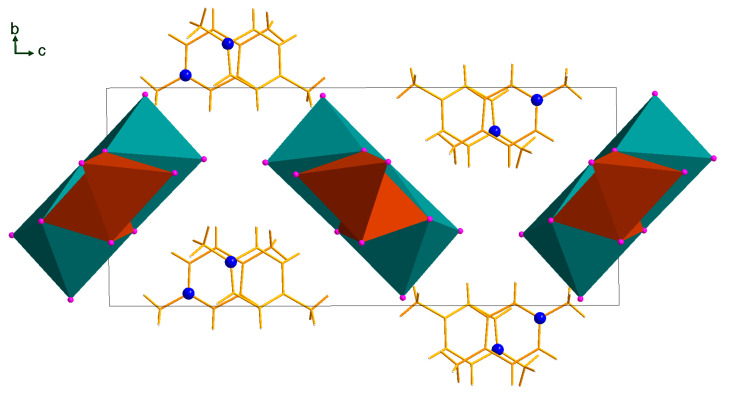
Crystal packing along *a* axis in **1**. [BiI_6_]^3−^ octahedra olive, [CuI_4_] tetrahedra brown. N blue, C yellow, I on the polyhedral vertices purple.

We previously reported on three compounds (**2**, **8**, and **9**) [41,42]; all others were synthesized using a similar approach (see below) and characterized via XRD methods. The addition of EtOH to a 1:1 acetone/CH_3_CN mixture leads to the formation of Bi/Cu heterometallic coordination polymers. While having the same formula and a similar polymeric anionic motif, the compounds crystallize in different crystal systems (either triclinic or monoclinic). The {[Cu_2_Bi_2_I_10_]_n_}^2n−^ anion was previously described in relevant heterometallic iodobismuthates [21,25]. Monomeric counterparts for this one-dimensional coordination polymer are known (see Figure S1), including [Bi_2_M_2_I_12_]^2−^ copper- and silver-containing iodobismuthates [19,33] and numerous complexes containing {L_2_Bi_2_M_2_I_12_} units [21,24,25,31]. The {[Cu_2_Bi_2_I_10_]_n_}^2n−^ coordination polymer can be described as a {Bi_2_I_10_} unit sharing its four-terminal and two μ_2_–iodide ligands with Cu atoms, thus forming a one-dimensional anionic polymeric motif (Figure 2). Therefore, the coordination sphere of Cu is composed of three μ_3_–iodide atoms and one μ_2_–iodide atom. The Bi-I_term_ bond lengths lie within a range of 2.89–2.92 Å; M–Iμ_2_ and M–Iμ_3_ distances are 2.63–2.65 and 2.62–2.65 Å for M = Cu, and 3.01–3.05 and 3.16–3.33 Å for M = Bi, respectively. The Cu···Cu distances are 2.73–2.89 Å. Heterometallic chains in compounds **1**–**4** are oriented along the *a* axis (Figure 3).

**Figure 4:**
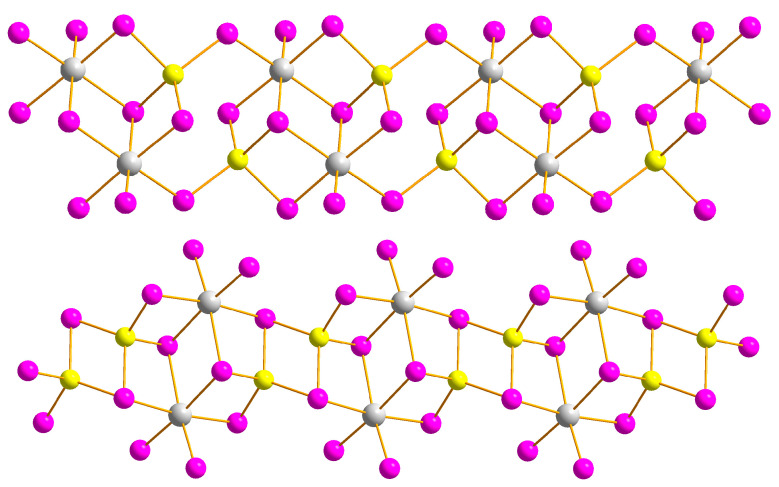
Anionic parts of compounds **5**–**7** [{β-Bi_2_Ag_2_I_10_}_n_]^2n−^ (**top**) in comparison with [{α-Bi_2_Ag_2_I_10_}_n_]^2n−^ of compound **8** (**bottom**). Bi grey, I purple, Ag yellow.

**Figure 5:**
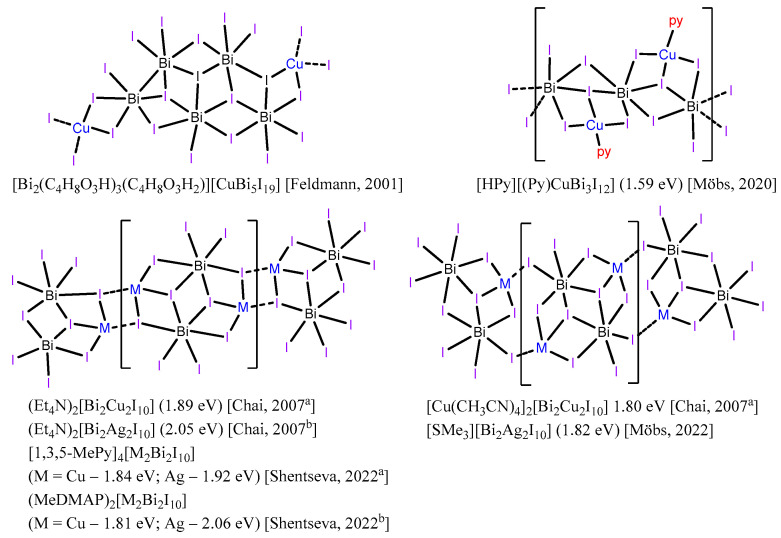
Overview of one-dimensional anions of copper and silver iodobismuthates and their band gaps in eV taken from literature (Feldmann, 2001 [20]; Chai, 2007 [25]; Chai, 2007 [26]; Möbs, 2020 [28], Möbs, 2022 [27]; Shentseva, 2022 [41], Shentseva, 2022 [42]). In compound from [20] (**top left**), the band gap for copper iodobismuthate was not provided.

By changing the heterometal and using silver(I) iodide instead of copper(I) iodide, we succeeded in isolating four heterometallic Ag^+^-containing iodobismuthates. In this series of experiments, it was noted that the addition of ethanol plays an ambiguous role, helping us obtain heterometallic phases in compounds **5**–**7**, or hindering their formation in the cases of compounds **8** and **9**. The anions of compounds **5**–**9** are polymeric, featuring the same [Bi_2_M_2_I_10_]^2−^ building units as in compounds **1**–**4**, but there are some important structural variations. The connectivity patterns in compounds **5**–**9** are different. The [{Bi_2_Ag_2_I_10_}_n_]^2n−^ anion of compound **8** closely resembles the analogous Cu-containing species in compounds **1**–**4** (Figure 4, top), with the Ag coordination sphere built of three μ_3_–iodide atoms and one μ_2_–iodide atom. In compound **8**, the Bi–I_term_ bond lengths are 2.89–2.90 Å; the M–μ_2_–I and M–μ_3_–I distances are 2.78 and 2.84–2.88 Å for M = Ag, and 3.07 and 3.15–3.33 Å for M = Bi, respectively. In contrast, the anions of compounds **5**–**7** are built of tetranuclear fragments {Bi_2_Ag_2_I_10_}, which are connected via two μ_2_–iodide ligands (Figure 4, bottom). Therefore, the coordination environment of Ag atoms consists of three μ_2_–iodide ligands and one μ_3_–bridging iodide ligand. Disordering Ag atoms with equal occupancies of positions in compound **9** was highlighted earlier [41], and a similar feature can be found in compound **7**, as the Ag atoms are split into two positions with occupancies of 0.96 and 0.04. The Ag···Ag distances in compounds **5**–**7** are 4.59–4.60 Å, which is 54% more than in compound **8** (2.99 Å). The Bi–I_term_, Bi–Iμ_2_ and Bi–Iμ_3_ distances in compounds **5**–**7** are 2.89–2.90, 3.05–3.14 and 3.13–3.35 Å, respectively, while the {Ag–I} are 2.79–2.87 and 2.92 Å for M–Iμ_2_ and M–Iμ_3_, respectively. In the case of compound **7**, Ag atoms with an occupancy of 0.94 were considered for distance measurements. Detailed structural data for compounds **1** and **3**–**7** are given in Supplementary Materials (Table S1). All prepared compounds belong to two previously known structural motifs. A comparison of the structural types of one-dimensional copper and silver iodobismuthates is presented in Figure 5. The most common isomer of [{Bi_2_Ag_2_I_10_}_n_]^2n−^ is represented by six compounds [25,26,41,42], while the least common types are represented by only one example each [20,28].

**Figure 6:**
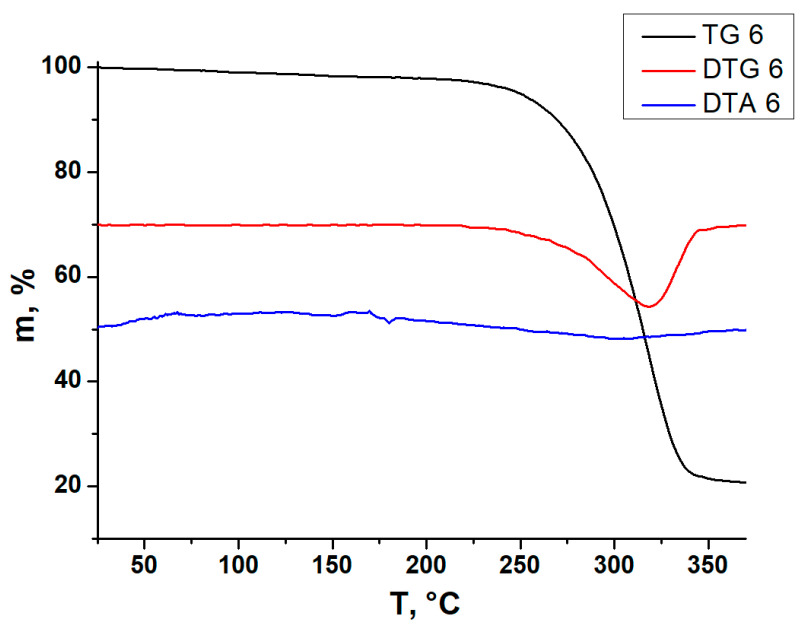
TG (black), DTG (red) and DTA (blue) curves for compound **6**.

All compounds are air-stable for at least several weeks and, according to PXRD data (see Supplementary Materials, Figures S2–S7), were obtained as single phases. This property enables further characterization of solid samples by using other physical methods, including thermogravimetric analysis (TGA). TGA was carried out for the whole series of compounds **1**–**9**. Full details are given in Supplementary Materials. It can be noted that all abovementioned compounds are stable at least up to 200 °C. Interestingly, these values are lower than those we previously reported [42] for the most thermally stable Bi/Cu and Bi/Ag hybrids with 1-MeDMAP^+^ cations (compounds **2** and **8**, respectively). Their decomposition temperatures are about 300 and 280 °C for compounds **2** and **8**, respectively. This was highlighted in earlier work [42] and is consistent with the stability of known discrete [M(bipy)_3_]-templated copper and silver iodobismuthates [33,34] or similar one-dimensional polymers, which are stable up to 200 °C with an anomaly for [Cu(CH_3_CN)_4_]_2_)[Cu_2_Bi_2_I_10_] caused by the elimination of coordinated CH_3_CN at 80 °C [25,26]. The TG curves for compounds **1**,**3**–**5** and **7** are given in Supplementary Materials (Figures S8–S11), while those for compound **6**, selected as the representative example within this series, are shown in Figure 6. Relatively high thermal stability is quite common for iodometallates with quarternized ammonium cations, so these data agree well with the literature. This feature fully meets one of the criteria for the use of such compounds in photovoltaic tests (overall, maximum thermal stability is desired).

**Figure 7:**
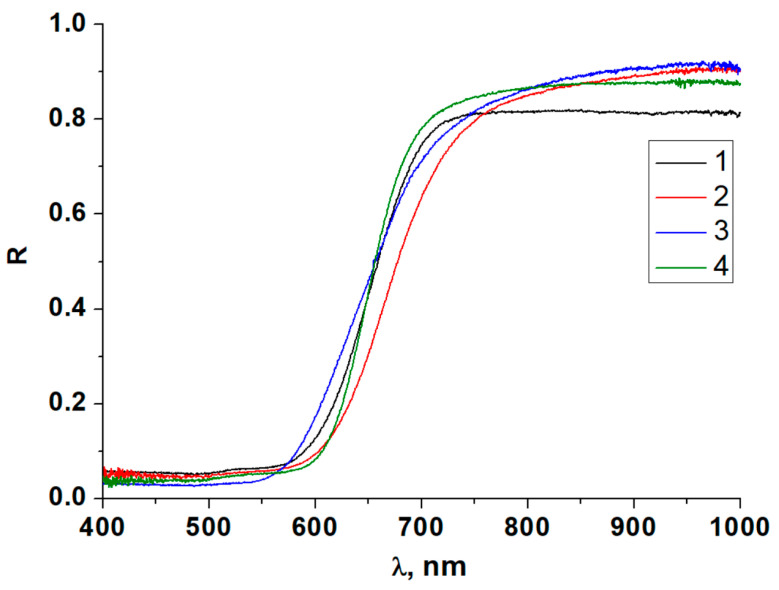
Diffuse reflectance spectra at room temperature for copper-containing iodometallates **1**–**4**.

**Figure 8:**
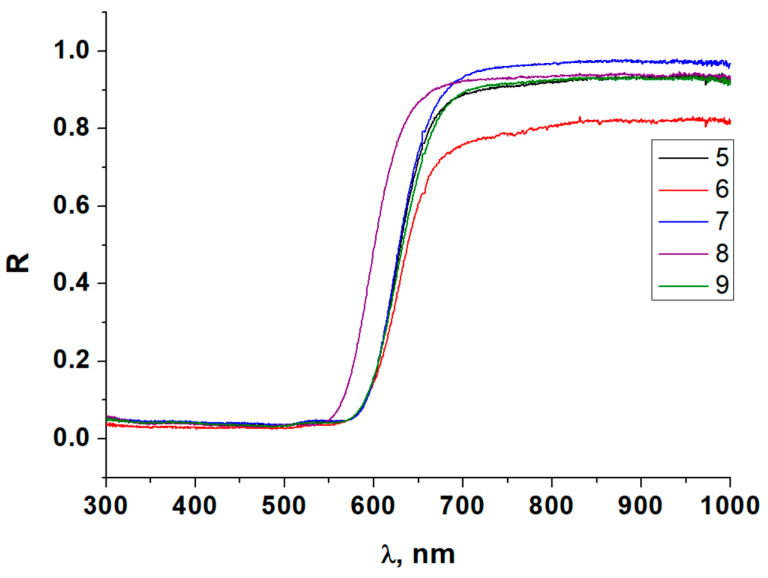
Diffuse reflectance spectra at room temperature for silver-containing iodometallates **5**–**9**.

As mentioned above, the primary goal of this work was to examine optical properties. Diffuse reflectance spectroscopy is the most common method for such studies for both bulk phases and thin films. As a starting point, we measured the corresponding spectra at room temperature for compounds **1**–**9** (for some of the samples, these data were collected and reported earlier [41,42]). These spectra are demonstrated in Figure 7 and Figure 8. The overall shape of the spectra is very similar in all cases and agrees well with the literature data [40], but it can be seen that certain differences do appear, even for the compounds with identical structure and composition of the anionic parts. This effect, in our opinion, can be explained by differences in the systems of non-covalent interactions between the organic cations and iodometallate anions (mostly hydrogen bonds of diverse strength).

Through direct comparison of various related copper- and silver-containing iodobismuthates (Figure 5 and Figure S1 in Supplementary Materials) of different structural types, we can conclude that, as a rule, the band gap of Cu-containing substances is lower than that of silver iodobismuthates, except for (SMe_3_)_2_[Ag_2_Bi_2_I_10_] with an unusually small (1.82 eV) band gap [27]. The mean E_g_ value (at room temperature) calculated for Bi/Cu iodometallates **1**–**4** is lower than for Ag-containing hybrids **5**–**9** (1.85 vs. 1.97 eV), and these values are in a very good agreement with previously published data for polymeric (Et_4_N)_2_[Cu_2_Bi_2_I_10_] (1.89 eV [25]), (Et_4_N)_2_[Cu_2_Bi_2_I_10_] (2.05 eV [26]), as well as for discrete [PPh_4_]_4_[Cu_2_Bi_2_I_12_] and [PPh_4_]_4_[Ag_2_Bi_2_I_12_] (1.8 vs. 2.1 eV [19]) heterometallic compounds.

Many works on halide complexes dealing with thermochromism describe this feature in terms of color changes and luminescent behavior [43,44] or by comparing crystallographic parameters by taking into account the sets of X-ray diffraction data at two or more temperatures [35,36,39,45,46]. It should also be noted that two different thermochromic mechanisms have been highlighted in the literature previously: charge transfer decrease, which can be found in iodoplumbate/iodoargentate complexes [43,44,47], as well as the contraction of lattice parameters and subtle changes in bond lengths in more structurally labile iodobismuthates [36] or heterometallic Ag/Cu modified iodoplumbates [48]. The latter explanation is more frequently mentioned.

**Figure 9:**
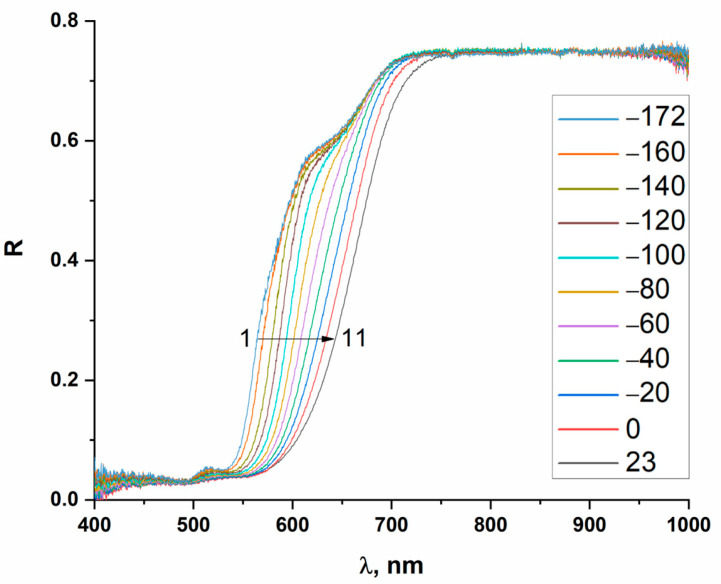
Diffuse reflectance spectra of compound **1** recorded from −172 °C (curve 1) to +23 °C (curve 11). Unmarked curves correspond to temperatures from −160 °C to 0 °C in 20 °C steps when moving from left to right.

To examine the temperature dependencies of optical spectra for compounds **1**–**9** (thermochromic behavior), we used the original setup we have previously described [40], which allows for multiple measurements of optical spectra during the cooling and heating of samples. (Figure 9).

**Figure 10:**
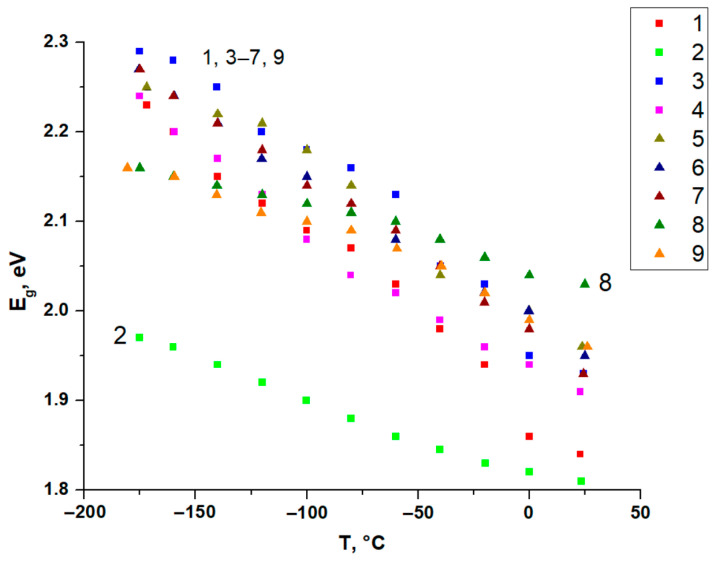
Temperature dependencies of E_g_ for compounds **1**–**9** in range of −170 °C to room temperature.

In most cases, the temperature dependencies E_g_ within compounds **1**–**9** are close to linear (Figure 10). The thermal coefficients for optical band gap (TCE_g_) are given in Table 1.

The largest ΔE_g_ equals 0.4 eV for compound **1**, which is much higher than for 2-methylimodasolium iodobismuthate [38], (0.04 eV) and lower than the 0.74 redshift for iodoargentate complex with 4-cyanopyridinium [49]. Interestingly, the values of TCE_g_ vary in a rather wide range—from −0.00066 for compound **7** to −0.00197 for compound **1,** with an average value of −1.42 meV/°C. This observation differs from that previously reported for bromotellurate(IV) complexes [40], where the maximal difference between TKE_g_ within a series of 16 complexes did not exceed 0.7 meV/°C, with an average value −1.26 meV/°C. In our opinion, this is also due to the aforementioned non-covalent interactions between cations and anions, which do differ depending on the nature of the cation and its ability to form hydrogen bonds. Considering that it plays a role even in the case of structurally simple mononuclear [TeBr_6_]^2−^ salts [40], it could be expected that for more sophisticated anions, the influence of this factor may be more pronounced, such as the 1D polymers in compounds **1**–**9**. An indirect argument in favor of this suggestion is the fact that compounds **2** and **8** are isostructural. Spectral data at minimal and maximum temperatures and separate temperature dependencies for copper- (compounds **1**–**4**, Figures S12–S19) and silver-containing iodobismuthates (compounds **5**–**9**, Figures S20–S28) are given in Supplementary Materials.

## 3. Materials and Methods

All compounds were obtained from commercial sources and used as purchased. A series of substituted pyridine iodides (1,3,5–triMePy^+^, 1,4–diMePy^+^, 1,3–diMePy^+^, 1–MeDMAP^+^, 3–Cl–1–MePy^+^,3–Br–1–MePy^+^) was obtained via reaction of corresponding pyridine with CH_3_I in acetonitrile. The purity of the iodides was confirmed by 1H NMR measurements. Elemental analysis results for compounds **1**, **3**–**9** are given in Table 2. Compounds **2**, **8** and **9** were prepared according to procedures we have previously described [41,42].

### 3.1. Preparation of Compounds ***1***, ***3*** and ***4***

For our experiments, we used 30 mg (0.05 mmol) of BiI_3_, 9.5 mg (0.05 mmol) of CuI and an appropriate amount of organic cation iodide (0.05 mmol for compounds **1**, **3** and **4**), which were dissolved upon heating (70 °C, 1 h) in 5 mL (or 10 mL in cases of compounds **3** and **4**) acetonitrile/acetone mixture (ratio 1:1 by volume). After complete dissolution of reactants, 5 mL of EtOH was added to the mixture. Solutions were then slowly cooled to room temperature. After 24 h, dark-red crystalline precipitates formed in all cases. Yields for compounds were: 59% (**1**), 71% (**3**) and 68% (**4**).

### 3.2. Preparation of Compounds ***5***–***7***

For the following experiments, 30 mg (0.05 mmol) of BiI_3_, 12 mg (0.05 mmol) of AgI and 0.05 mmol of organic cation (12 mg of 1,3–diMePyI (**5**), 13 mg 3–Cl–1–MePyI (**6**) and 15 mg 3–Br–1–MePyI (**7**)) were dissolved in a mixture of acetonitrile/acetone (9, 12 or 13 mL for compounds **5**, **6** and **7,** respectively; ratio 1:1 by volume). After the complete dissolution of the reactants, 5 mL of EtOH was added to the mixture. The solution was then slowly cooled to room temperature. After 24 h, red crystals formed. Yields for compounds: 58% (**5**), 61% (**6**) and 56% (**7**).

### 3.3. Thermogravimetric Analysis

Thermogravimetric analyses (TGA) were carried out using a TG 209 F1 Iris thermobalance (NETZSCH, Bayern, Germany). The measurements were made in a helium flow in a temperature range of 30–450 °C, using a heating rate of 10 °C min^–1^ and a gas flow rate of 60 mL min^–1^. Open Al crucibles were used for measurements.

### 3.4. Diffuse Reflectance Spectroscopy

Diffuse reflectance spectra were measured on an original setup comprising a Kolibri-2 spectrometer (VMK Optoelektronica, Novosibirsk, Russia), a fiber optic probe FCR-7UVIR400-2-ME-HT and a deuterium–tungsten lamp AvaLight-DHS (Avantes, Apeldoorn, The Netherlands). BaSO_4_ powder served as a 100% reflectance reference. To perform measurements at different temperatures, both the sample and a Type-K thermocouple were placed in a closed vessel (with the thermocouple located on the surface of sample), which was mounted on a vertical rod above a dewar filled with liquid nitrogen. The temperature was regulated by movements of the dewar, which was placed on scissor jack. For each sample, 18 to 22 spectra were measured in a range from −170 to 25 °C; the error decreased as temperature increased (from 4 to 1 °C). A detailed description of the experimental setup can be found in previous work [40].

### 3.5. Single-Crystal X-ray Diffraction Data Collection

The single crystal X-ray diffraction data for **3** and **5**–**7** (Table S1) were collected with a Bruker D8 Venture diffractometer with a CMOS PHOTON III detector and IμS 3.0 source (Mo Kα radiation, λ = 0.71073 Å, φ- and ω-scans). Data reduction was performed routinely via Apex3 suite (Apex3, SADABS 2016/2 and SAINT 8.40a; Publisher: Bruker AXS Inc., Madison, WI, USA, 2017.). The data for **1** and **4** were collected with an Agilent Xcalibur diffractometer (Agilent, Santa Clara, CA, USA) equipped with an area AtlasS2 detector (graphite monochromator, λ(MoKα) = 0.71073 Å, ω-scans) (Procentec, Wateringen, The Netherlands. Data reduction was performed routinely via the CrysAlisPro program package v1.

The crystal structures were solved by dual space algorithm by SHELXT, 2015 [50] and refined by the full-matrix least squares technique by SHELXL [51] with Olex2 GUI (2009) [52]. Atomic displacements for non-hydrogen atoms were refined in harmonic anisotropic approximation. Hydrogen atoms were located geometrically and refined in a riding model. For the structure of **7**, Ag atom showed uncorrelated ADP and was disordered over two positions with the occupancy of 96/4%. The structure of **1** was refined as a 2-component twin with the ratio of 94/6; this partially solved the problem of high residual electron density. In the case of **4**, the high residual electron density is an artefact arising from somewhat poor quality of the crystal. The structures of **1**–**7** were deposited to the Cambridge Crystallographic Data Centre (CCDC) as a supplementary publication, No. 2244394–2244399.

### 3.6. Powder X-ray Diffraction Data Collection

Powder X-ray diffraction data for polycrystalline samples were collected with Shimadzu XRD-7000 diffractometer in the Bragg—Brentano geometry (CuKα radiation, Ni–filter) and Bruker Advance powder diffractometer with an energy discriminating Eyger XE T detector (CuKα radiation). The samples were slightly ground with hexane in an agate mortar, and the resulting suspensions were deposited on the polished side of a standard sample holder, and a smooth thin layer being formed after drying. The diffraction patterns of **1**–**7** agree well with those simulated from the single crystal XRD data.

## 4. Conclusions

We successfully obtained a series of Cu- and Ag-containing heterometallic iodobismuthates with different pyridinium-based cations and studied their thermochromic behavior. Thermochromism was found to be a common feature for compounds **1**–**9**, but the dependence of the optical band gap on temperature varied significantly. We hypothesize that these differences may be attributed to variations in cation/anion non-covalent interactions within crystalline lattices. As far as we know, our report is the first systematic study of thermochromic dependencies for heterometallic Bi/Cu and Bi/Ag compounds (and is also among the few works on iodobismuthates(III) of this type). These data could be of significant importance for further development of photovoltaic materials, since it is quite unclear how changes in device temperature, which occur during actual operation, may affect the performance of a photovoltaic cell.

## Figures and Tables

**Table 1:** Temperature dependencies of E_g_ for compounds **1**–**9** (TKE_g_ = dE_g_/dT).

Compound	TKE_g_, meV/°C	Compound	TKE_g_, eV/°C
**1**	−1.97	**6**	−1.56
**2**	−0.86	**7**	−1.66
**3**	−1.88	**8**	−0.66
**4**	−1.67	**9**	−0.93
**5**	−1.59		

**Table 2:** Data from element analysis for compounds **1**, **3**-**7**.

Compound	C, H, N, Calculated/Found, %	Compound	C, H, N, Calculated/Found, %
**1**	8.3, 1.0, 1.4/8.7, 1.2, 1.2	**5**	7.9, 1.0, 1.3/8.1, 1.2, 1.6
**3**	7.0, 0.7, 1.4/7.4, 1.0, 1.5	**6**	6.7, 0.7, 1.3/7.0, 1.3, 1.4
**4**	8.3, 1.0, 1.4/8.1, 1.2, 1.4	**7**	6.4, 0.6, 1.2/6.9, 1.0, 1.4

## Data Availability

XRD data were deposited in the Cambridge structural database (codes 2244394–2244399).

## Supplementary Materials

The following supporting information can be downloaded at: https://www.mdpi.com/article/10.3390/ijms24087234/s1.
