# Supplementary material for: Copper- and Silver-Containing Heterometallic Iodobismuthates: Features of Thermochromic Behavior
Source: Int J Mol Sci. 2023 Apr 13;24(8):7234. doi: 10.3390/ijms24087234 (PMC10138349; doi:10.3390/ijms24087234)
Supplement: Supplementary file 1 [file ijms-24-07234-s001.zip › ijms-2275622-supplementary.pdf]

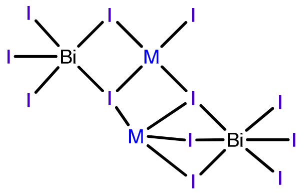

$[PPh_4]_4[M_2Bi_2I_{12}]$
(Cu - 1.8 eV; Ag - 2.1 eV)
[Dehnhardt, 2018]
$[Fe(bipy)_3]_2[Ag_2Bi_2I_{12}]$ (1.92eV)
[Zhang, 2022[a]]
$[Ni(bipy)_3]_2[Ag_2Bi_2I_{12}]$(2.13 eV)
[Zhang, 2022[a]]

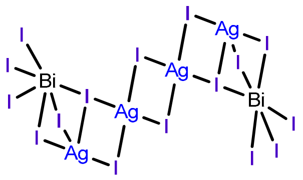

$[Co(bipy)_3]_2[Ag_4Bi_2I_{16}]$
(1.95 eV) [Zhang, 2022[b]]

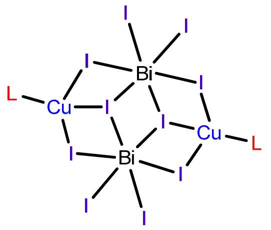

$(L)_2Bi_2Cu_2I_{10}$ (1.82 eV)
L = $EtDABCO^+$ [Cai, 2021]
$[PBz_4]_2[(L)_2Bi_2Cu_2I_{10}]$
L = CH_3CN (2.06 eV) [Dehnhardt, 2020]
$[n\text{-}Bu_4N]_2[L_2Cu_2Bi_2I_{10}]$
L = CH_3CN (2.06 eV) [Chai, 2007[a]]
$[Bu_4N]_2[(L)_2Bi_2Cu_2I_{10}]$
L = PPh_3 (3.44 eV*) [Kelly, 2017]
L = $P(OPh)_3$ (3.49 eV*) [Kelly, 2017]

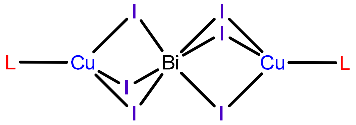

$[(HL)][BiI_6(CuL)_2]$
(L = $P(o\text{-}tol)_3$, 1.91 eV) [Möbs, 2021]

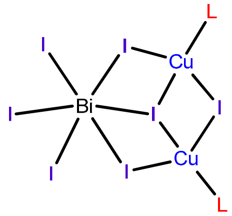

$[(L)_2BiCu_2I_7](C_2H_5OH)$
L = $DABCOH^+$, 1.91 eV) [Cai, 2021]
$[PPh_4]_2[(L)BiCu_2I_7]$
L = C_2H_5CN, (1.80 eV) [Dehnhardt, 2019]

Figure S1. Recent progress in discrete copper and silver iodobismuthates: known structural motifs of anions and estimated band gaps for compounds provided from literature (Dehnhardt, 2018 [19]; Dehnhardt, 2020 [21]; Dehnhardt, 2023 [23]; Kelly, 2017 [24]; Chai, 2007[a] [25]; Cai, 2021 [31]; Möbs, 2021 [32]; Chang, 2022[a] [33]; Chang, 2022[b] [34]). In [24] band gap was determined via DFT calculations instead of optical measurements.

Table S1. Crystal data and structure refinement for the compounds.

Identification code	**1**	**3**	**4**	**5**	**6**	**7**
CCDC number	2244394	2244395	2244396	2244397	2244398	2244399
Empirical formula	$C_{14}H_{20}Bi_2Cu_2I_{10}N_2$	$C_{12}H_{14}Bi_2Cl_2Cu_2I_{10}N_2$	$C_{14}H_{20}Bi_2Cu_2I_{10}N_2$	$C_{14}H_{20}Ag_2Bi_2I_{10}N_2$	$C_{12}H_{14}Ag_2Bi_2Cl_2I_{10}N_2$	$C_{12}H_{14}Ag_2Bi_2Br_2I_{10}N_2$
Formula weight	2030.36	2071.19	2030.36	2119.02	2159.85	2248.77
Temperature/K	150(2)	150(2)	130(2)	150(2)	150(2)	150(2)
Space group	*P*–1	$P2_1/c$	*P*–1	$P2_1/n$	$P2_1/n$	$P2_1/n$
a/Å	8.1532(3)	8.1843(3)	8.1160(4)	8.4937(3)	8.4917(3)	8.5108(2)
b/Å	10.0476(3)	15.5548(5)	10.1659(6)	14.4970(5)	14.4710(5)	14.4603(3)
c/Å	22.6474(11)	14.6497(5)	11.4804(6)	15.0043(5)	14.8197(6)	14.9074(3)
α/°	89.936(3)	90	99.852(4)	90	90	90
β/°	87.272(3)	104.0220(10)	96.634(4)	95.4670(10)	94.413(2)	94.9910(10)
γ/°	74.289(3)	90	105.844(5)	90	90	90
Volume/Å^3	1783.81(12)	1809.41(11)	884.53(9)	1839.12(11)	1815.70(12)	1827.68(7)
Z	2	2	1	2	2	2
ρ_{calc}g/cm^3	3.780	3.802	3.812	3.827	3.951	4.086
μ/mm^{-1}	19.674	19.542	19.838	18.991	19.382	21.295
F(000)	1744.0	1776.0	872.0	1816.0	1848.0	1920.0
Crystal size/mm^3	0.15 × 0.12 × 0.10	0.15 × 0.08 × 0.08	0.3 × 0.2 × 0.2	0.06 × 0.03 × 0.02	0.15 × 0.10 × 0.10	0.10 × 0.10 × 0.08
Radiation	MoKα (λ = 0.71073)	MoKα (λ = 0.71073)	MoKα (λ = 0.71073)	MoKα (λ = 0.71073)	MoKα (λ = 0.71073)	MoKα (λ = 0.71073)
2Θ range for data collection/°	4.212 to 58.122	3.882 to 66.278	6.788 to 50.052	3.916 to 63.032	5.358 to 72.784	3.932 to 72.948
Index ranges	-11 ≤ h ≤ 11, -13 ≤ k ≤ 13, -3 ≤ l ≤ 30	-12 ≤ h ≤ 12, -23 ≤ k ≤ 18, -16 ≤ l ≤ 22	-9 ≤ h ≤ 8, -9 ≤ k ≤ 12, -13 ≤ l ≤ 12	-12 ≤ h ≤ 12, -21 ≤ k ≤ 21, -22 ≤ l ≤ 22	-13 ≤ h ≤ 14, -23 ≤ k ≤ 24, -24 ≤ l ≤ 23	-14 ≤ h ≤ 14, -23 ≤ k ≤ 24, -19 ≤ l ≤ 24
Reflections collected	7868	23933	5614	35281	36035	29761
Independent reflections	7868 [R_{int} = 0.0343, R_{sigma} = 0.0620]	6867 [R_{int} = 0.0601, R_{sigma} = 0.0494]	3124 [R_{int} = 0.0484, R_{sigma} = 0.0791]	6128 [R_{int} = 0.0435, R_{sigma} = 0.0303]	8798 [R_{int} = 0.0326, R_{sigma} = 0.0281]	8877 [R_{int} = 0.0314, R_{sigma} = 0.0316]
Data/restraints/parameters	7868/108/276	6867/0/138	3124/0/138	6128/0/138	8798/0/138	8877/6/148
Goodness-of-fit on F^2	1.154	1.145	1.035	1.061	1.081	1.034
Final R indexes [I>=2σ (I)]	R_1 = 0.0570, wR_2 = 0.1238	R_1 = 0.0260, wR_2 = 0.0660	R_1 = 0.0521, wR_2 = 0.1098	R_1 = 0.0234, wR_2 = 0.0440	R_1 = 0.0233, wR_2 = 0.0439	R_1 = 0.0251, wR_2 = 0.0430
Final R indexes [all data]	R_1 = 0.0671, wR_2 = 0.1297	R_1 = 0.0290, wR_2 = 0.0672	R_1 = 0.0614, wR_2 = 0.1177	R_1 = 0.0294, wR_2 = 0.0470	R_1 = 0.0269, wR_2 = 0.0449	R_1 = 0.0316, wR_2 = 0.0445
Largest diff. peak/hole / e Å^{-3}	6.69/-1.98	1.62/-1.15	4.24/-5.57	1.18/-2.02	2.00/-2.27	2.16/-1.72

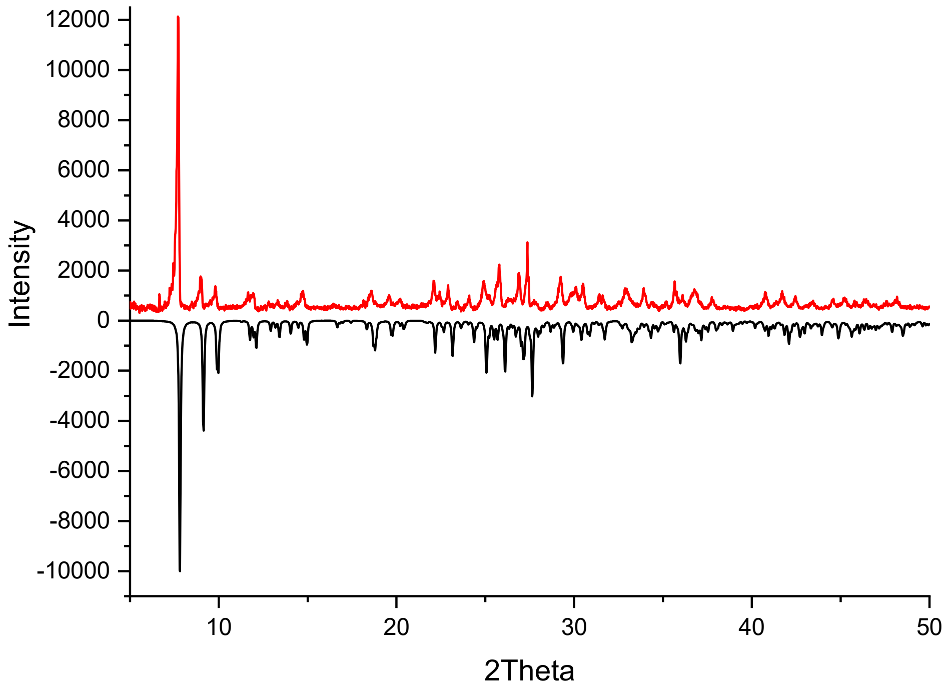

Figure S2. Powder XRD for the pure phase of **1** (*red*) shown in comparison with the pattern simulated from single crystal x-ray data for this compound (*black*).

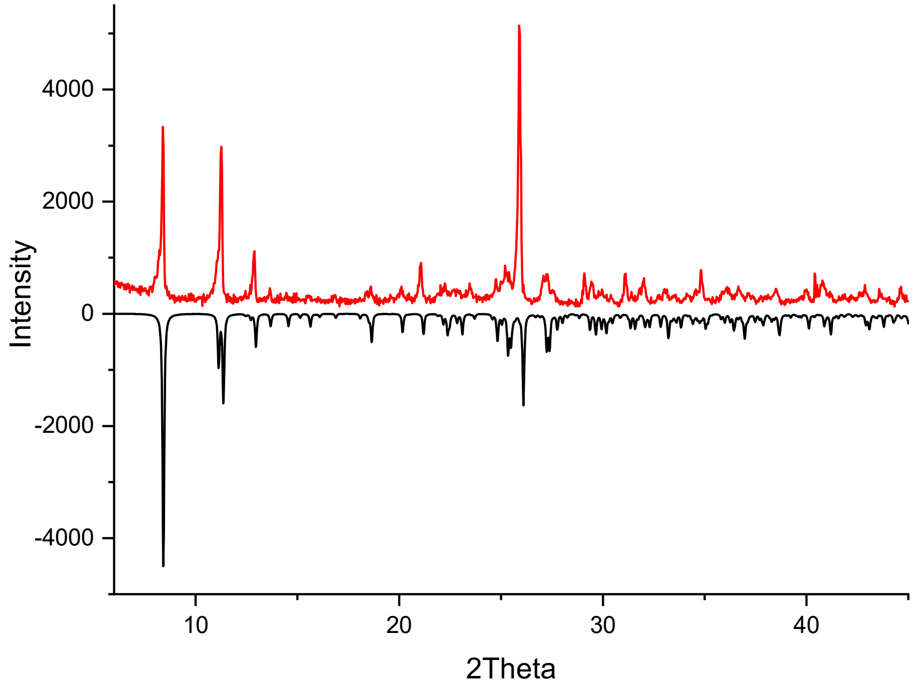

Figure S3. Powder XRD for the pure phase of **3** (*red*) shown in comparison with the pattern simulated from single crystal x-ray data for this compound (*black*).

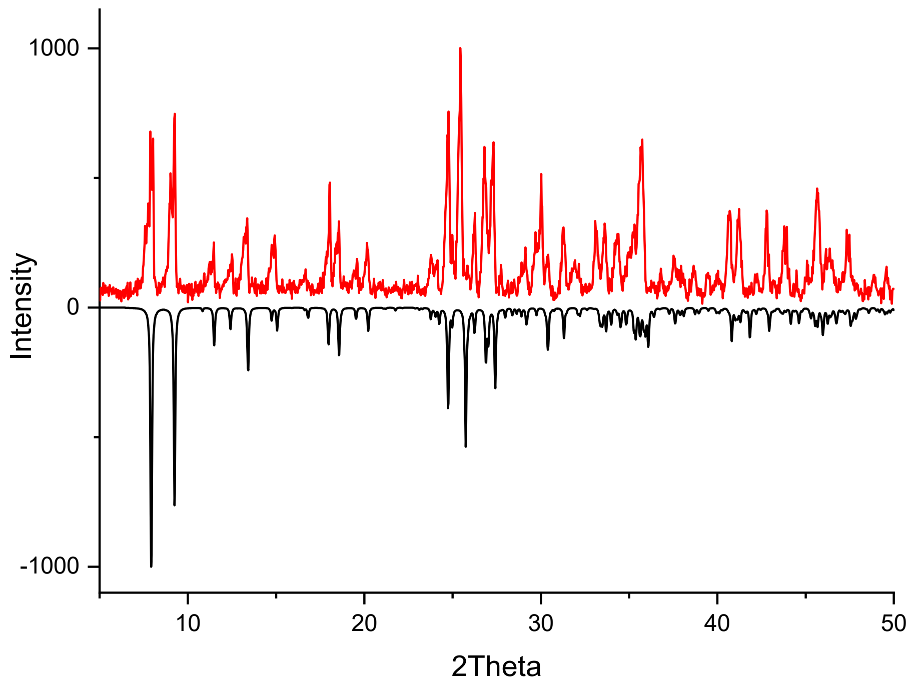

Figure S4. Powder XRD for the pure phase of **4** (*red*) shown in comparison with the pattern simulated from single crystal x-ray data for this compound (*black*).

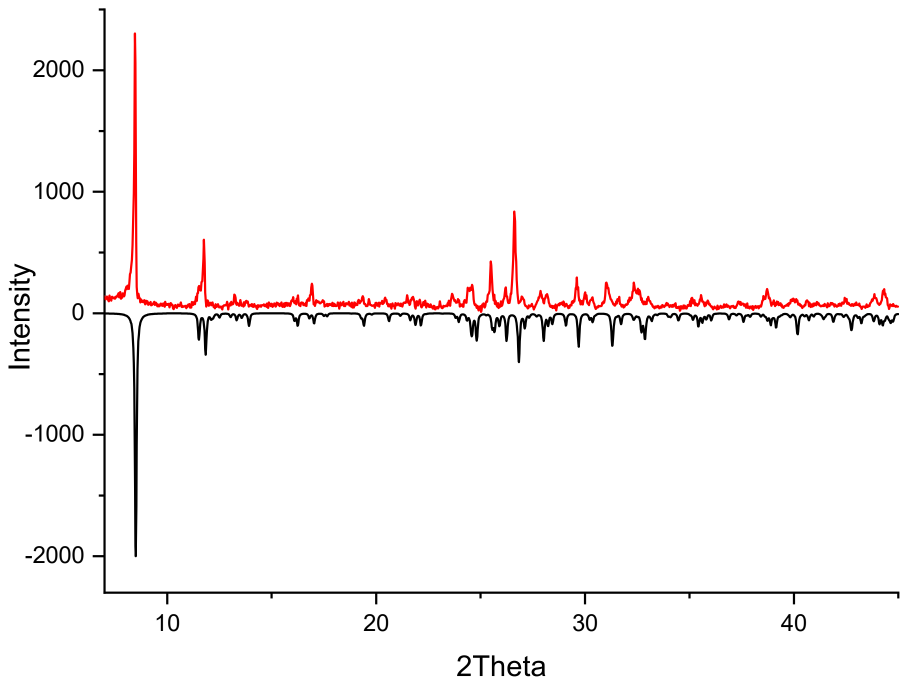

Figure S5. Powder XRD for the pure phase of **5** (*red*) shown in comparison with the pattern simulated from single crystal x-ray data for this compound (*black*).

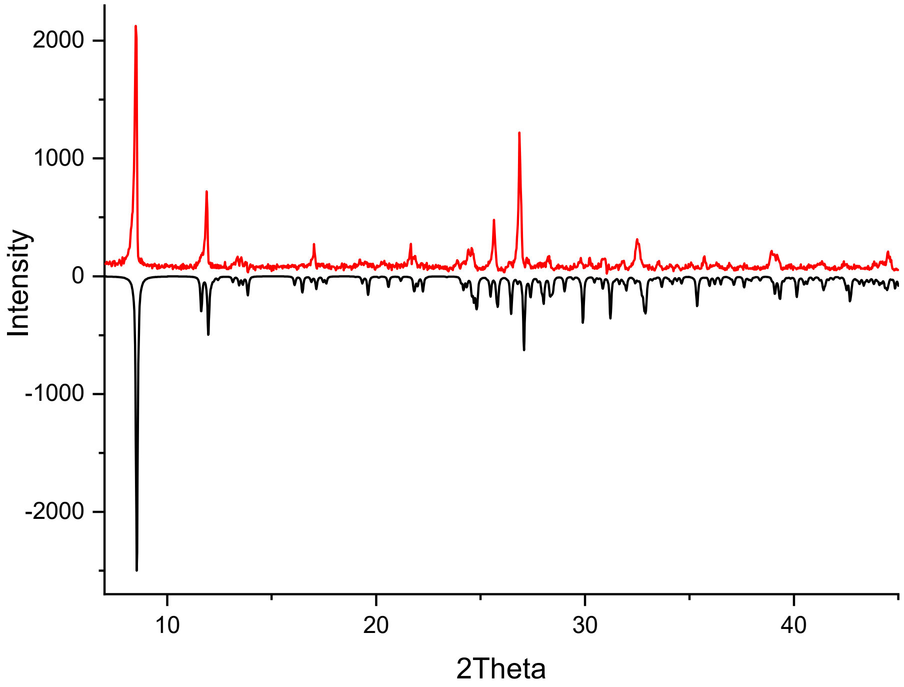

Figure S6. Powder XRD for the pure phase of **6** (*red*) shown in comparison with the pattern simulated from single crystal x-ray data for this compound (*black*).

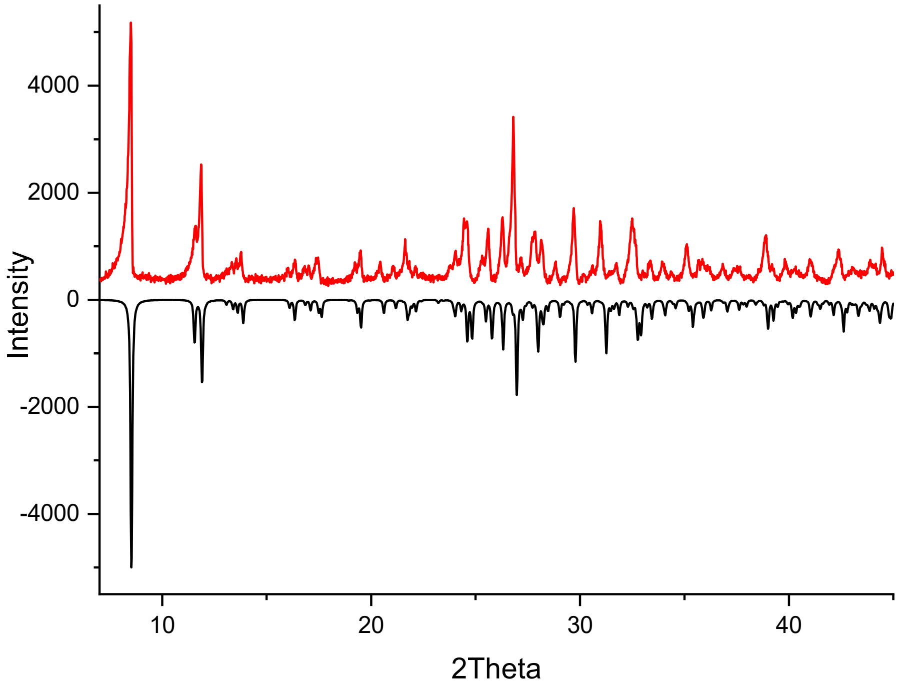

Figure S7. Powder XRD for the pure phase of **7** (*red*) shown in comparison with the pattern simulated from single crystal x-ray data for this compound (*black*).

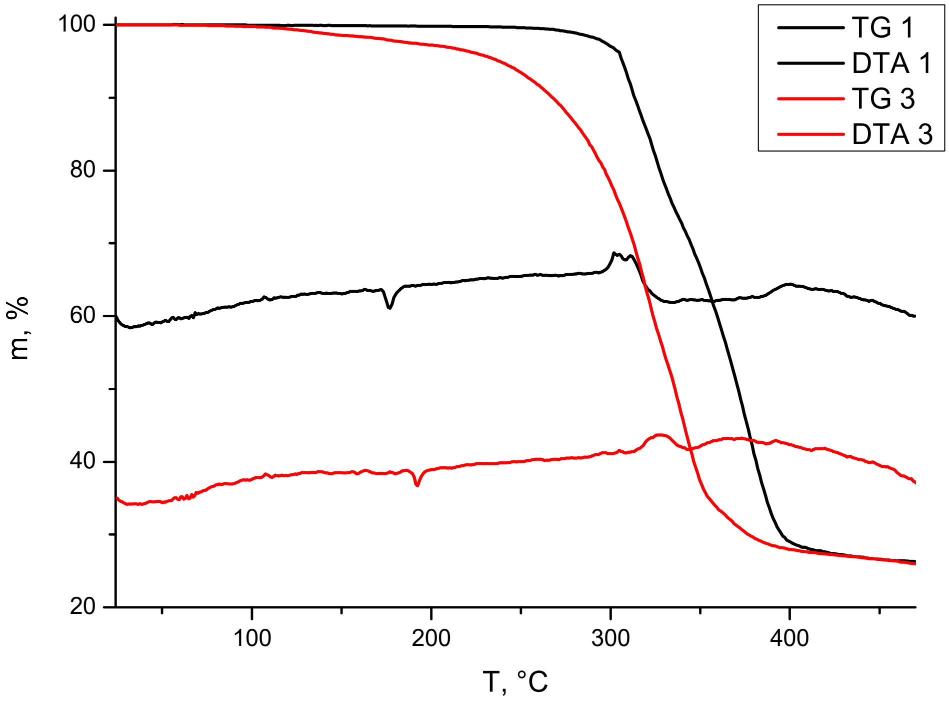

Figure S8. TG and DTA curves for **1** (*black*) and **3** (*red*).

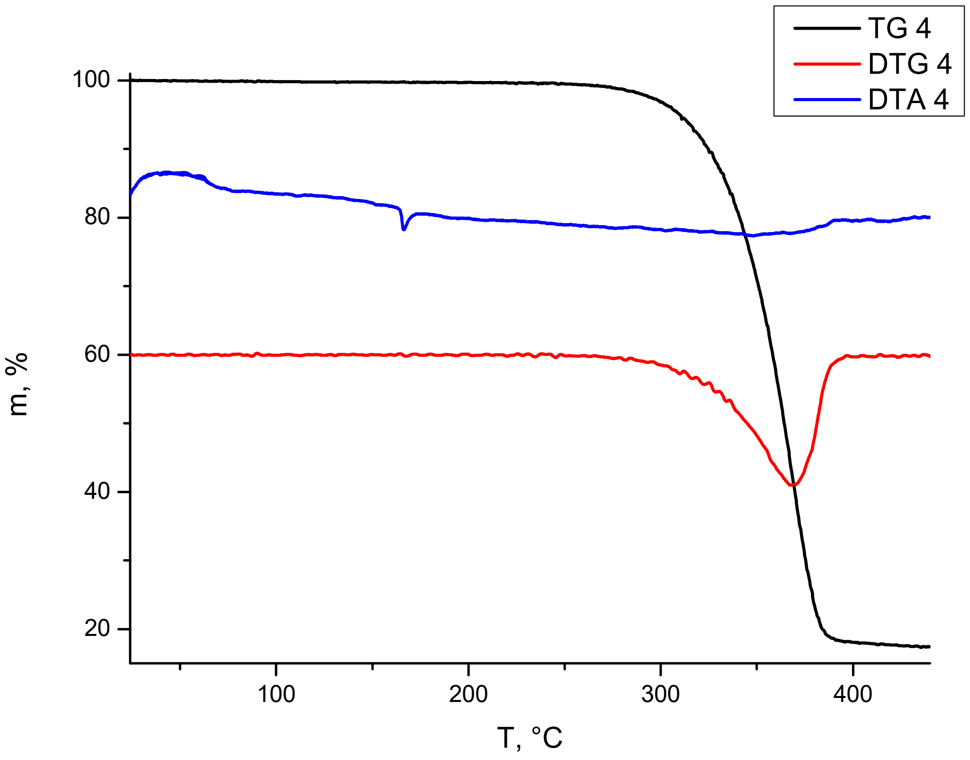

Figure S9. TG, DTG and DTA curves for **4**.

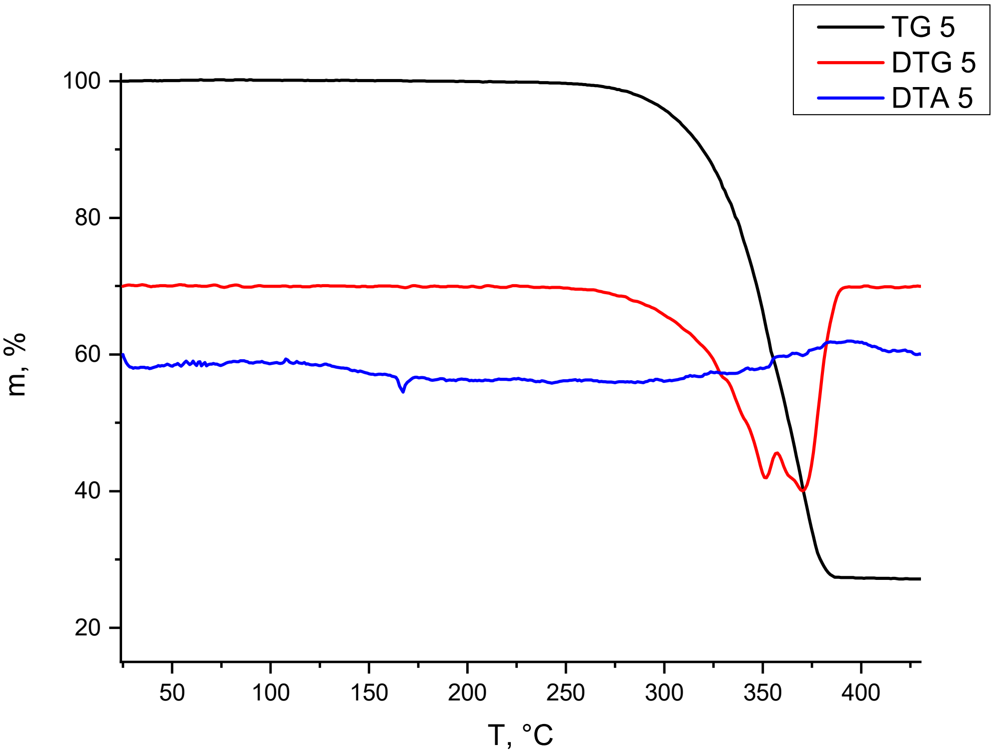

Figure S10. TG, DTG and DTA curves for **5**.

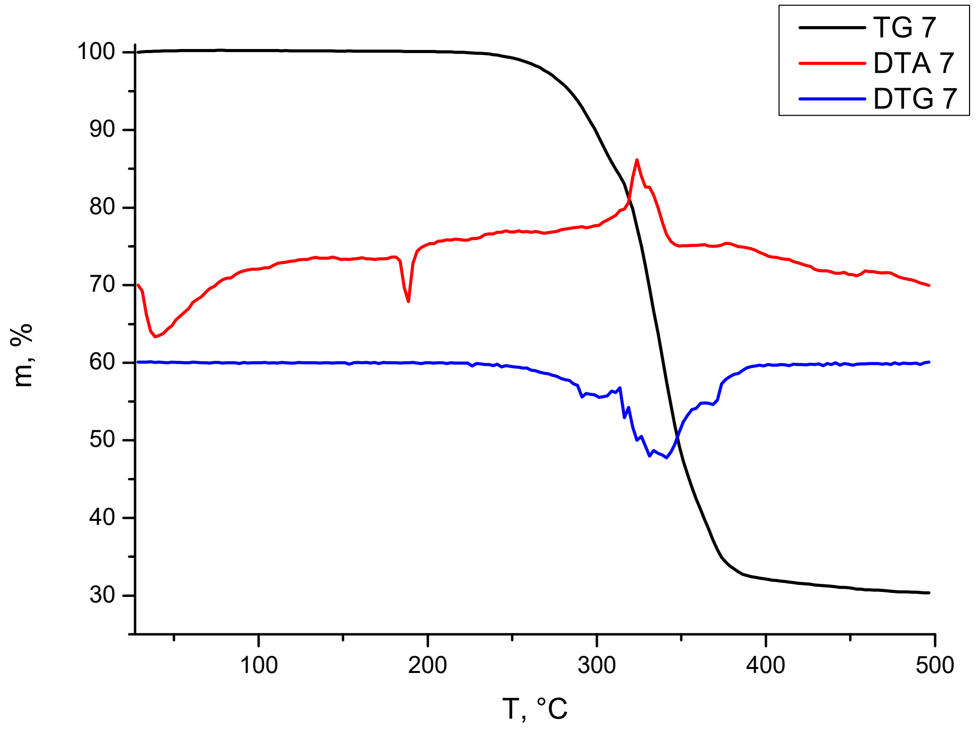

Figure S11. TG, DTG and DTA curves for **7**.

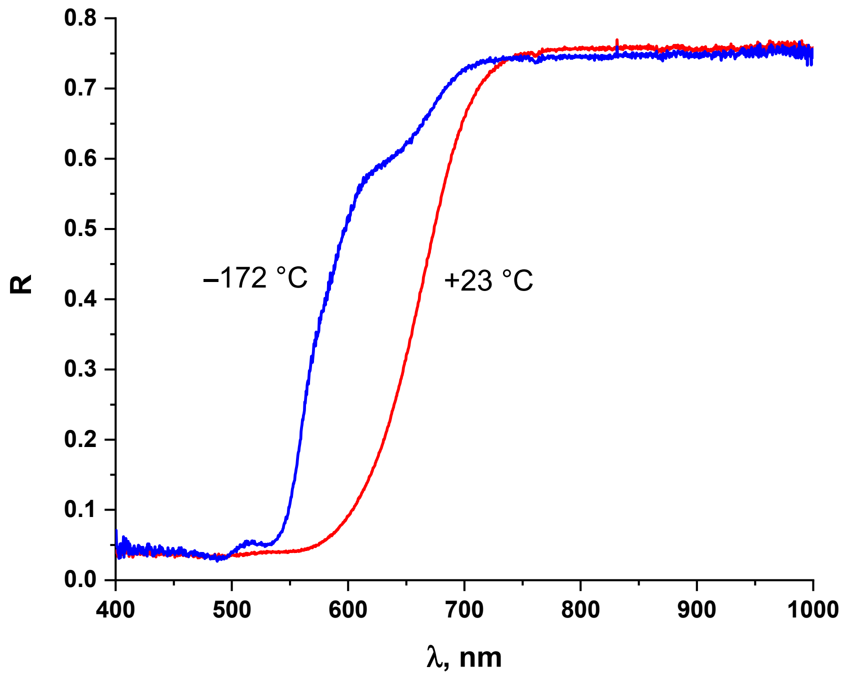

Figure S12. Diffuse reflectance spectra for **1**.

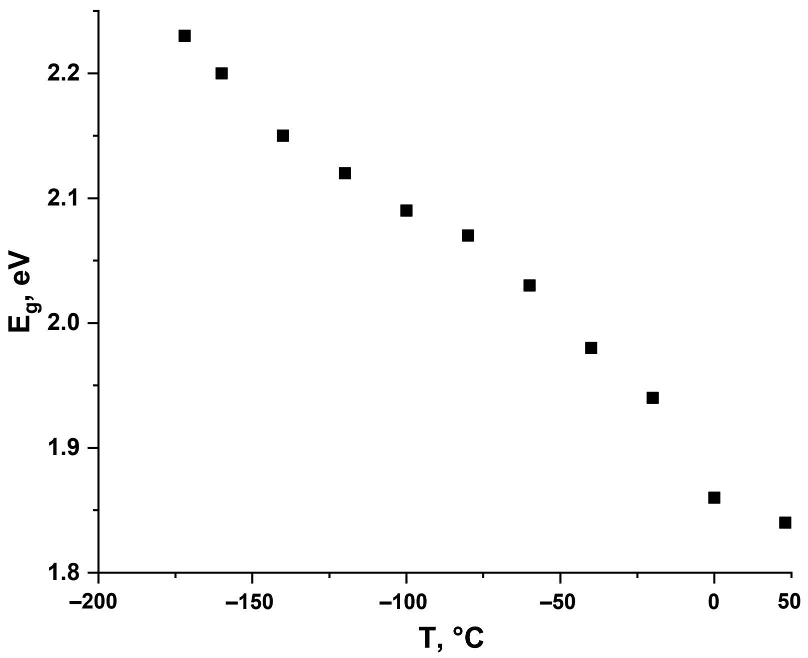

Figure S13. Temperature dependence of E_g for **1**.

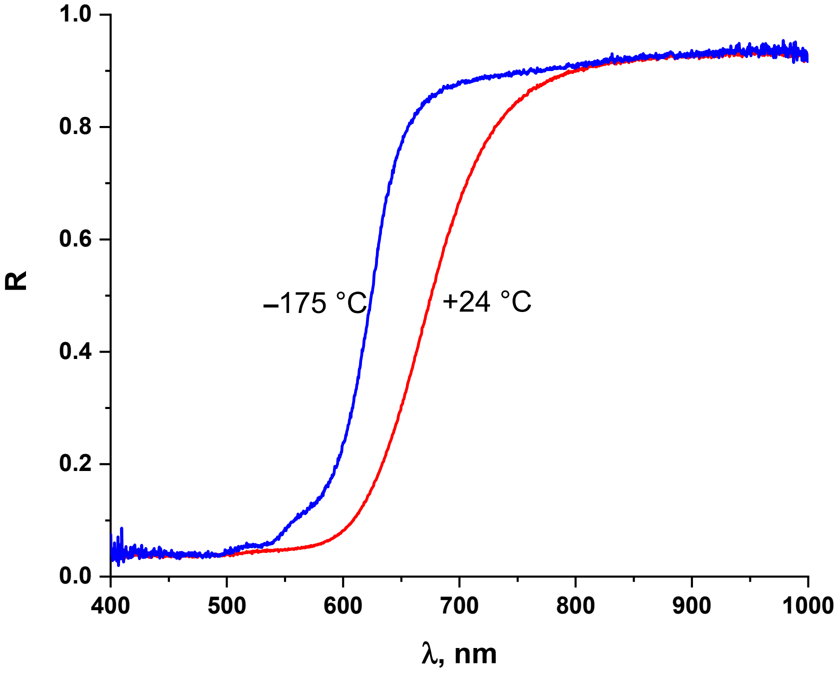

Figure S14. Diffuse reflectance spectra for **2**.

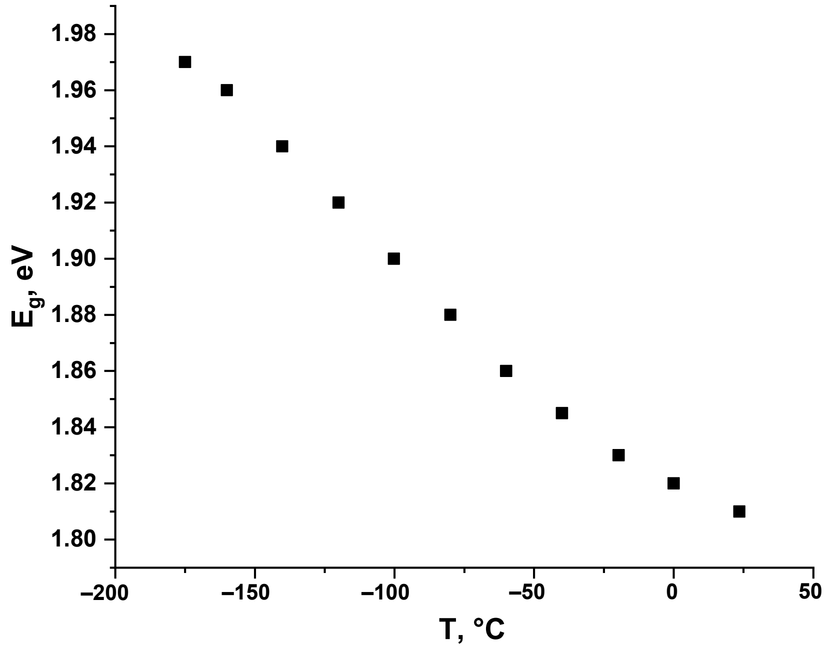

Figure S15. Temperature dependence of E_g for **2**.

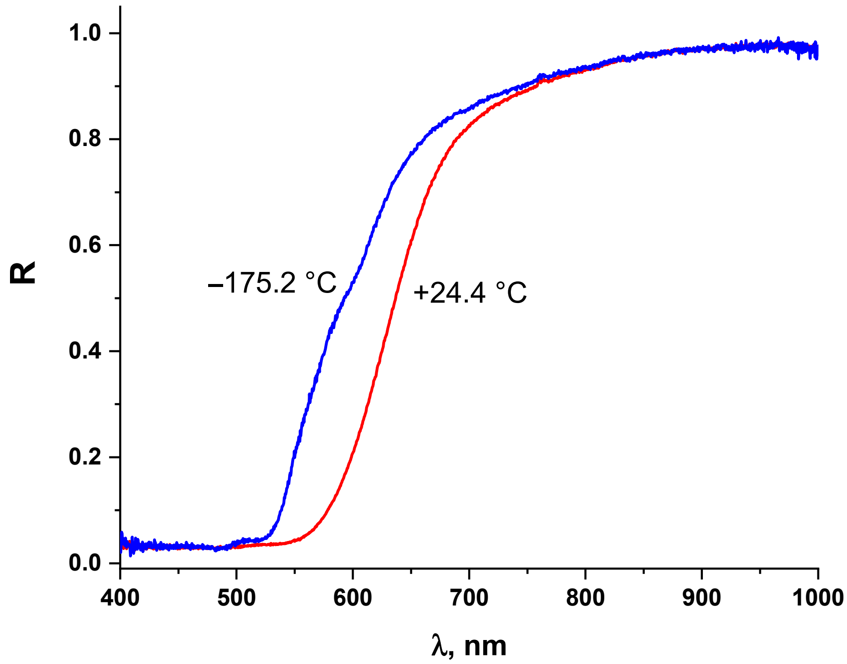

Figure S16. Diffuse reflectance spectra for **3**.

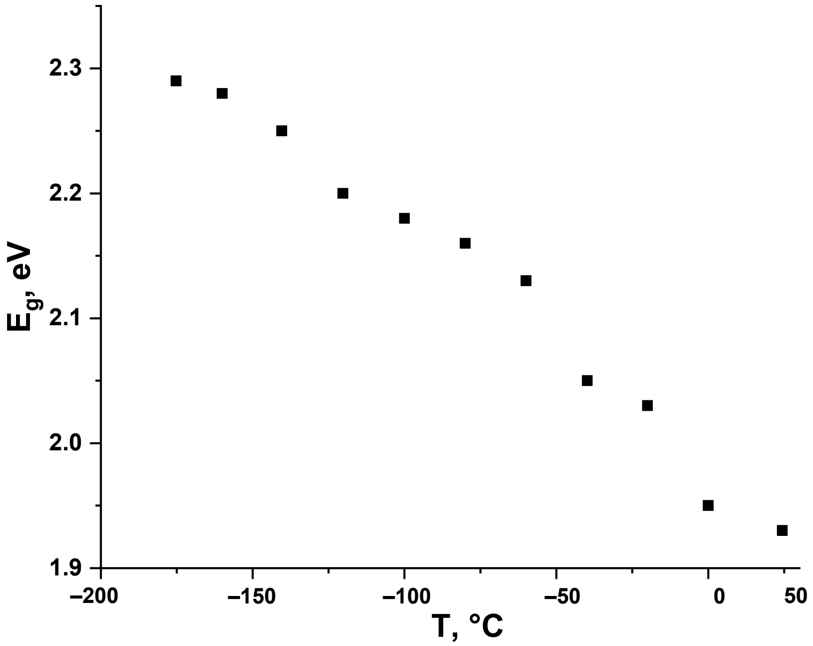

Figure S17. Temperature dependence of E_g for **3**.

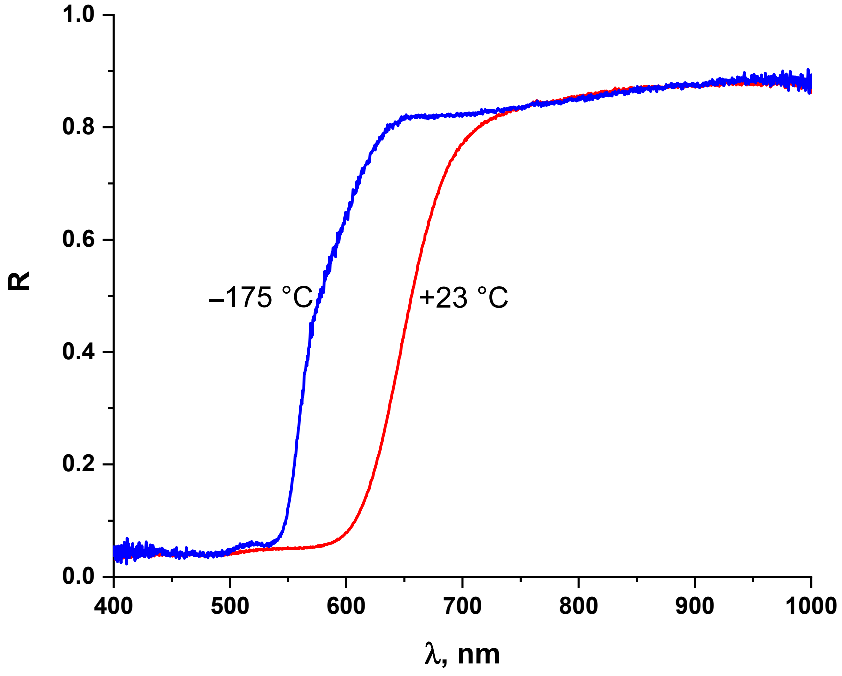

Figure S18. Diffuse reflectance spectra for **4**.

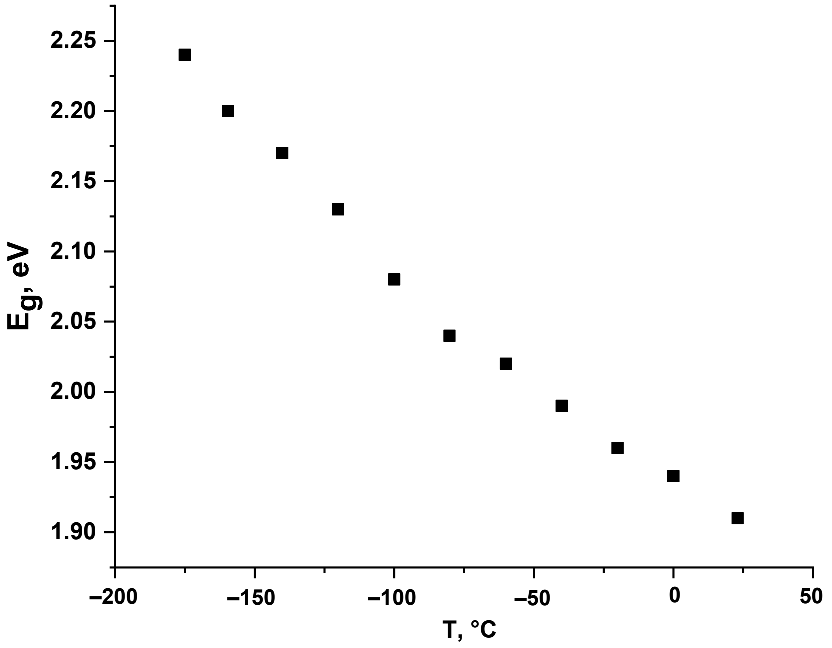

Figure S19. Temperature dependence of E_g for **4**.

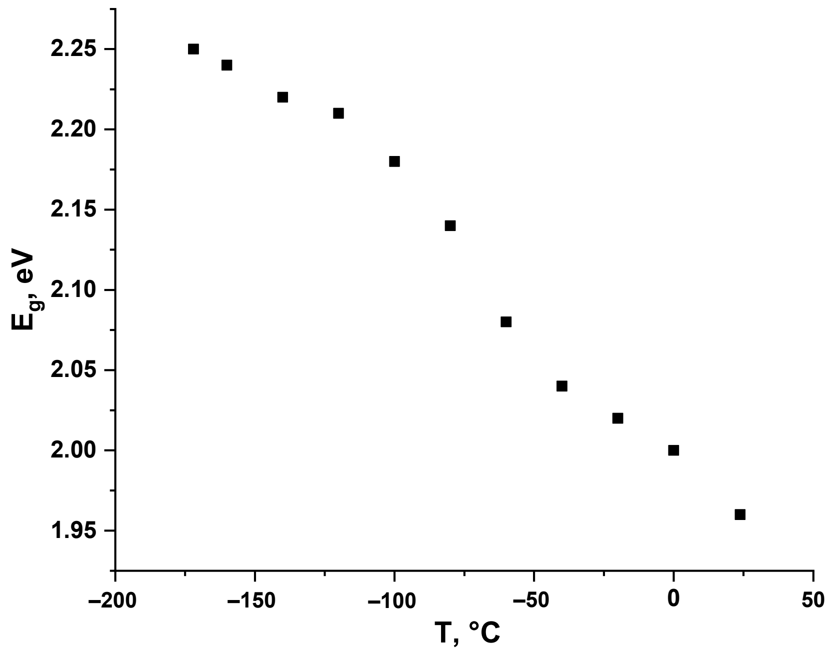

Figure S20. Temperature dependence of E_g for **5**.

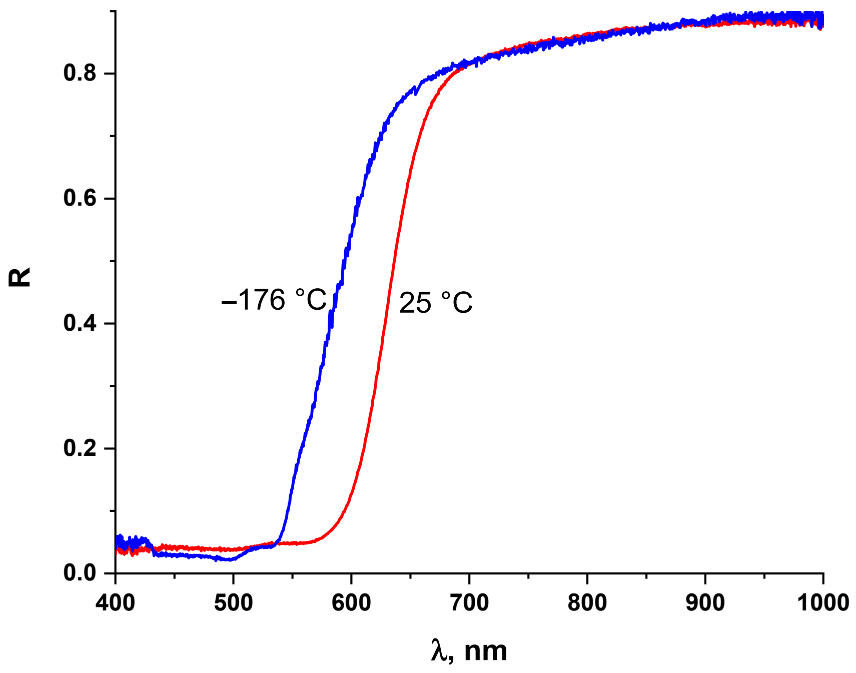

Figure S21. Diffuse reflectance spectra for **6**.

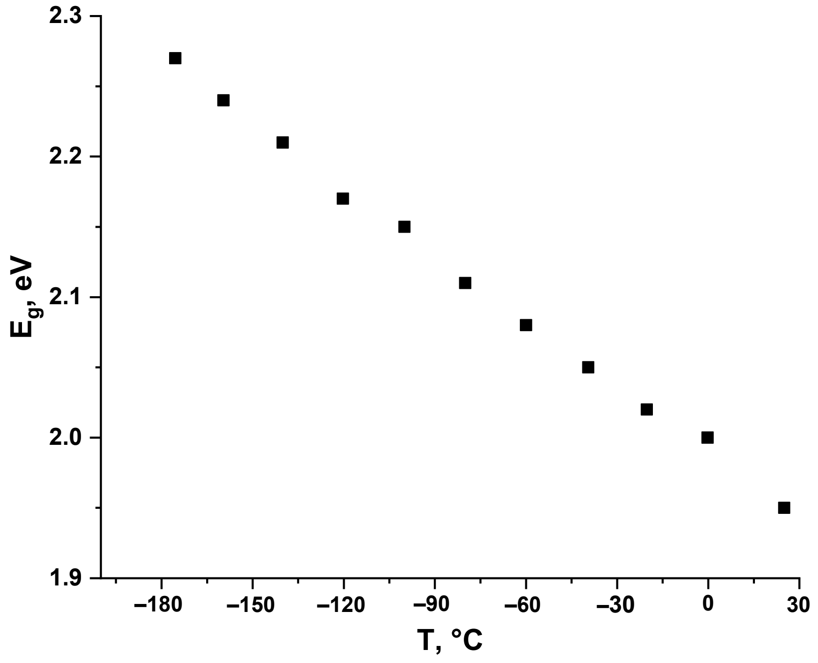

Figure S22. Temperature dependence of E_g for **6**.

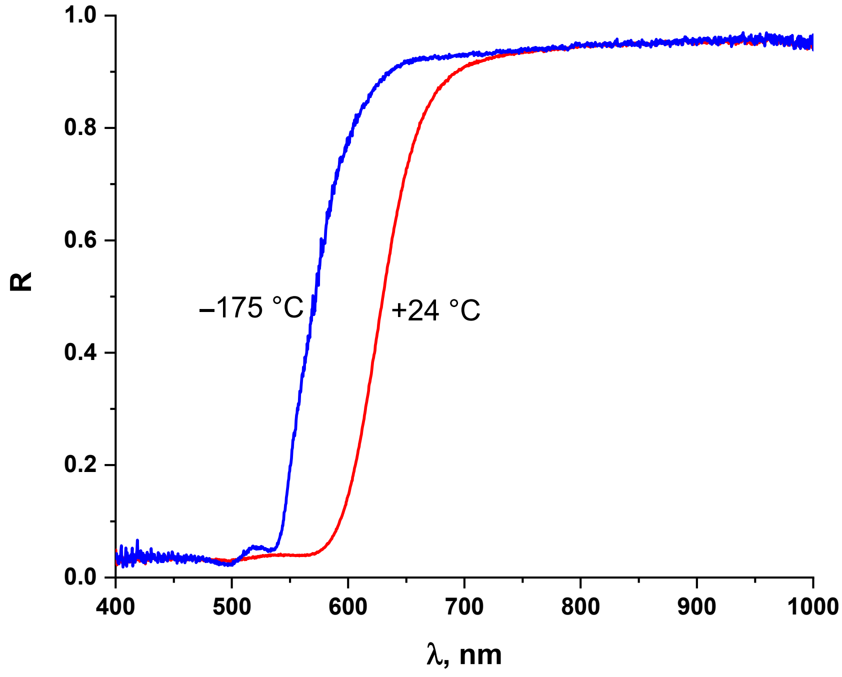

Figure S23. Diffuse reflectance spectra for **7**.

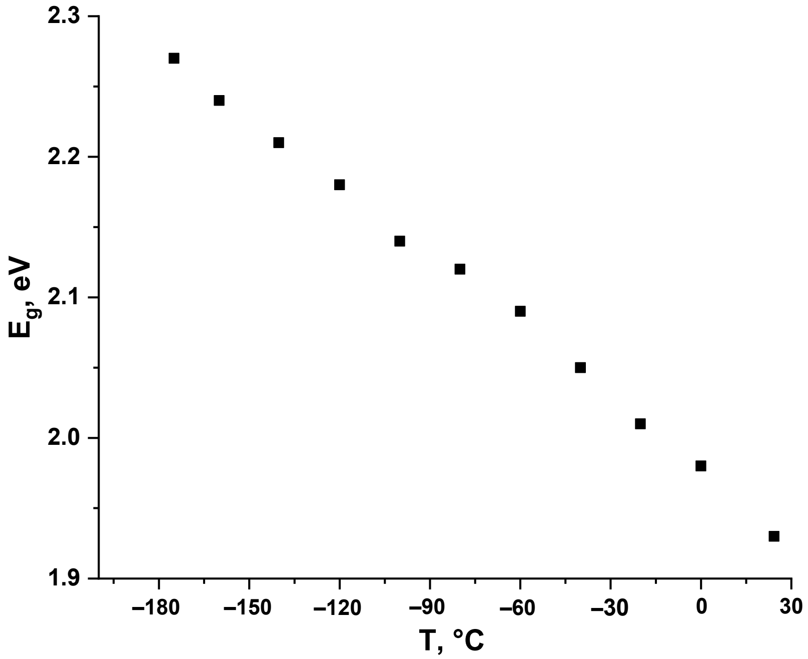

Figure S24. Temperature dependence of E_g for **7**.

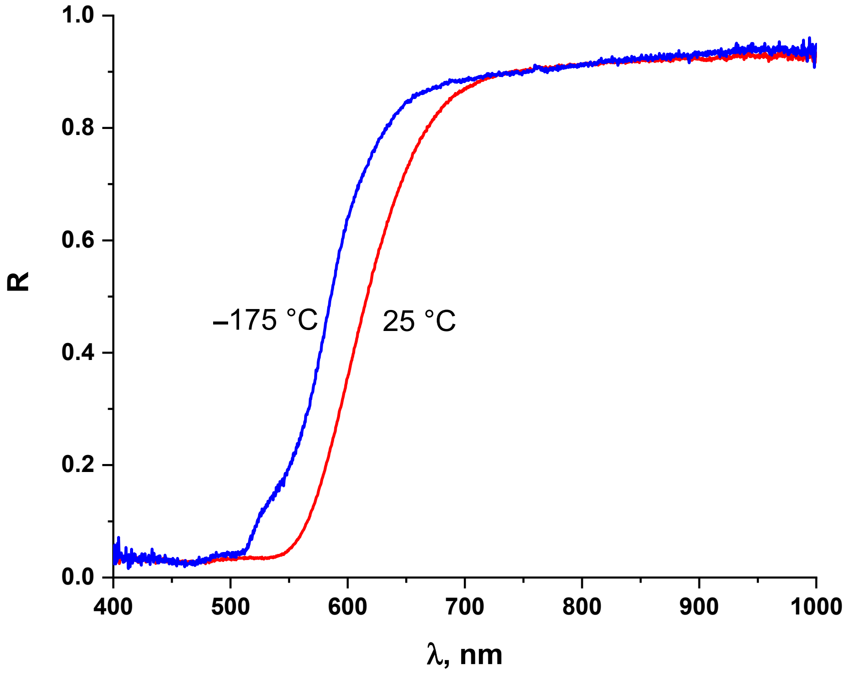

Figure S25. Diffuse reflectance spectra for **8**.

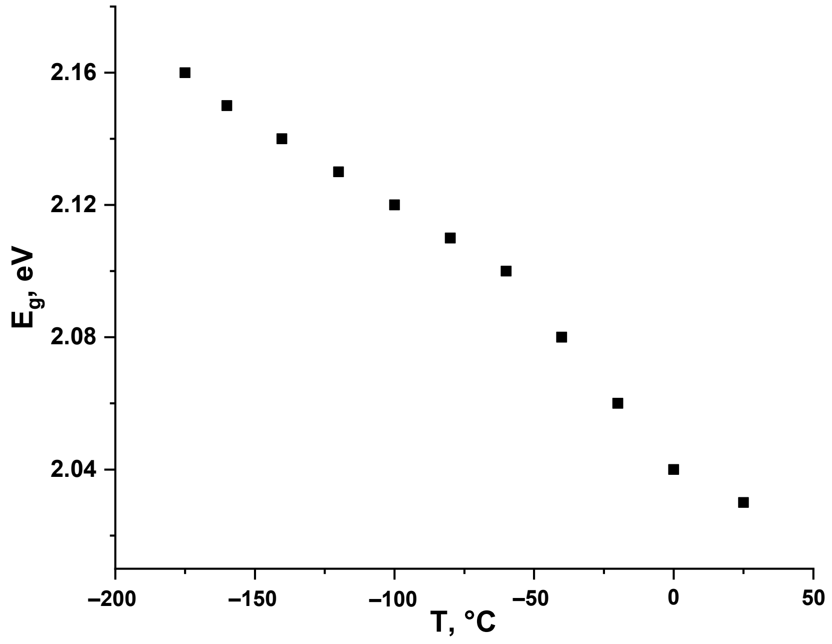

Figure S26. Temperature dependence of E_g for **8**.

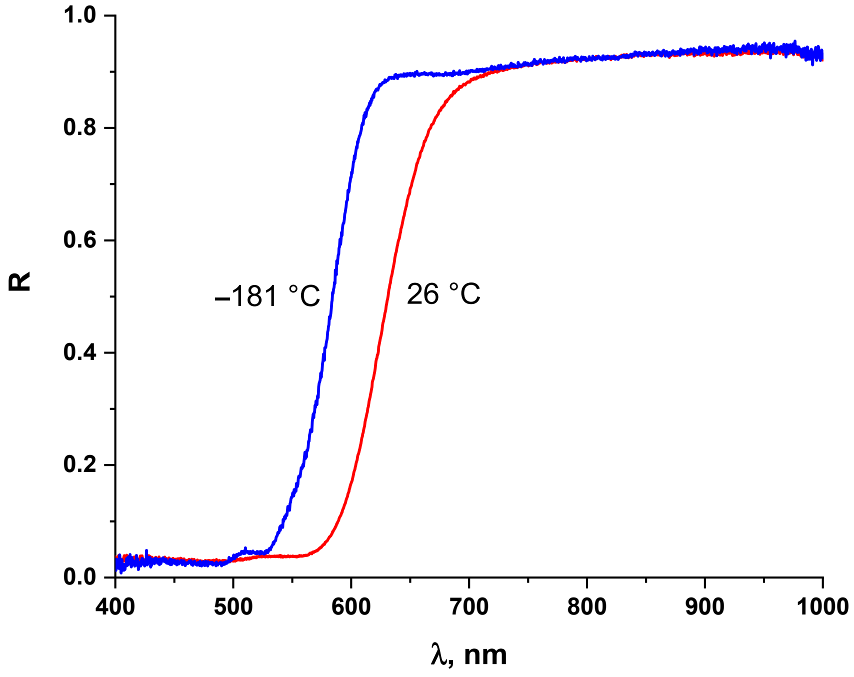

Figure S27. Diffuse reflectance spectra for **9**.

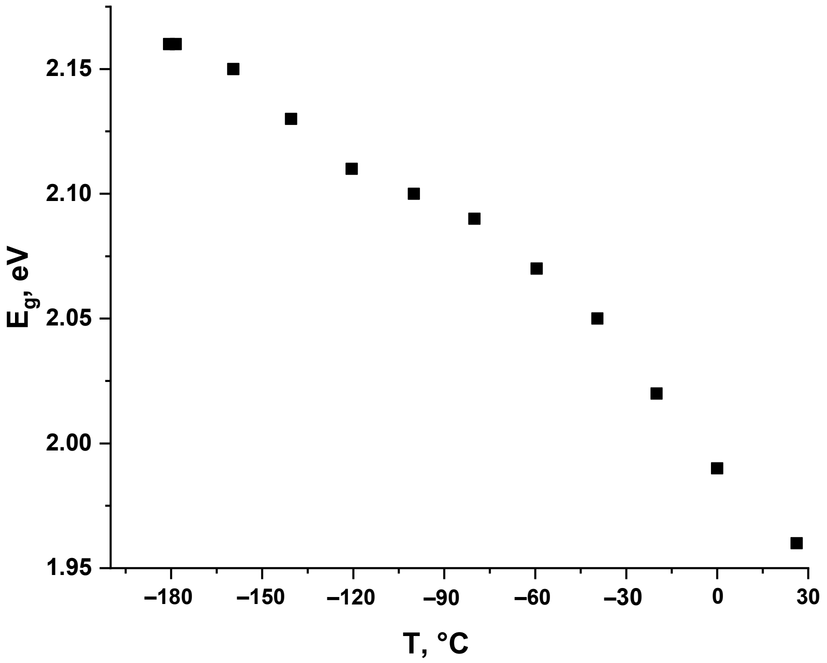

Figure S28. Temperature dependence of E_g for **9**.
